# Decreased antimicrobial prescribing rate following academic detailing of resident physicians in outpatient clinic

**DOI:** 10.1017/ash.2022.259

**Published:** 2023-03-31

**Authors:** Shannon L. Andrews, Amy A. Gravely, Amanda Beaudoin, Meghan K. Rothenberger, Dimitri M. Drekonja

**Affiliations:** 1 Department of Pediatrics, University of Minnesota, Minneapolis, Minnesota; 2 Minneapolis Veterans’ Affairs Health Care System, Minneapolis, Minnesota; 3 One Health Antibiotic Stewardship, Minnesota Department of Health, St. Paul, Minnesota; 4 Infectious Disease Section, Minneapolis VA Health Care System, Minneapolis, Minnesota; 5 Department of Medicine, University of Minnesota, Minneapolis, Minnesota; 6 Chief, Infectious Disease Section, Minneapolis Veterans’ Affairs Health Care System, Minneapolis

## Abstract

Among 37 internal-medicine resident physicians assigned to our outpatient clinic at Minneapolis Veterans’ Affairs Health Care System (MVAHCS) on July 1, 2017, we designed a pre- and postintervention observational study. Our results show that in-person academic detailing around outpatient antimicrobial selection was associated with a decrease in outpatient antimicrobial prescriptions in a group of high-prescribing resident physicians.

Antimicrobial stewardship is an organized response to antimicrobial overprescribing. The Centers for Disease Control and Prevention (CDC) estimate that 50% of antimicrobial prescriptions are inappropriate and that 30% are completely unnecessary.^1^ Most antimicrobial prescribing in the United States occurs in the outpatient setting.^1^ Although the CDC has codified components of outpatient antimicrobial stewardship,^2^ the most effective way to implement them remains unclear.^3^

Education regarding antimicrobial prescribing early in medical training is recommended,^4^ although no studies have yet proven that this leads to changes in future practice.^4^ Antimicrobial stewardship takes up <1% of the curricula in training programs outside infectious diseases fellowships.^5^ Studies show antimicrobial-use knowledge gaps among resident physicians, with accuracy on knowledge quizzes ranging from 28% to 61%.^6–9^ Most resident physicians are interested in learning more about antimicrobials; one study showed that 90% of respondents desired more education.^7^ Resident physicians in certain settings may have limited opportunities to treat acute infections in outpatient continuity clinics.^9^ We hypothesized that a targeted educational intervention would lead to a decrease in outpatient antimicrobial prescribing by resident physicians in their continuity clinics.

## Methods

All internal medicine resident physicians assigned to outpatient continuity clinic as of July 1, 2017, at the Minneapolis Veterans’ Affairs Health Care System (MVAHCS) were contacted for study participation (n = 37). The preintervention (baseline) period was July 1, 2017, to March 31, 2018. The intervention period continued from April 1, 2018, to March 31, 2019. Data were censored when participants graduated from residency. No incentives were provided for participation in this study.

We extracted the number of antimicrobial prescriptions and continuity clinic visits per resident physician from the computerized patient record system. Data were deidentified and associated with a unique study identifier linking prescriptions to the clinical visit. A monthly calculation of total antimicrobial prescriptions per 1,000 patient visits was performed.

### Educational intervention

We determined the median antimicrobial rate during the preintervention period then divided resident physicians into high-prescribing (n = 20) and low-prescribing (n = 17) groups. The low-prescribing group received an e-mail with links to the CDC outpatient antibiotic treatment guidelines^10^ as well as an interactive simulation of an outpatient clinic visit where prescription of antimicrobials is discussed. The high-prescribing group received the same e-mail plus an in-person meeting with an infectious disease fellow. This meeting was ∼15 minutes in length, discussing 2 cases from each resident physician’s recent clinic patients: 1 exemplary and 1 constructive. When cases specific to the resident physician were not available, general outpatient cases were discussed. The CDC outpatient antibiotic treatment guidelines were reviewed and any questions from the resident physician were addressed.

### Statistical analysis

We tested the difference in trends before versus during the intervention for both groups using a 2-piecewise random coefficient model using PROC MIXED in SAS version 9.4 software (SAS Institute, Cary, NC). An interrupted time-series analysis was used to assess the overall level of antimicrobial prescribing and any change in the prescribing rate associated with the high-intensity or low-intensity educational intervention separately. The MVAHCS Institutional Review Board reviewed this project and deemed it exempt from review as a quality improvement project.

## Results

In total, 37 internal medicine resident physicians who rotate through the VA continuity clinic were eligible for participation as of July 1, 2017. Among these participants, 25 remained in residency until the completion of the project on March 31, 2019. Demographics are discussed separately.^10^

During the baseline period, 182 antimicrobial prescriptions were written, and an antimicrobial was prescribed during 155 of 3,588 clinic visits (4.3% of visits), resulting in an average antimicrobial prescribing rate of 51 per 1,000 patient visits. During the intervention period, 171 antimicrobials were prescribed. This represented 171 separate clinic visits, or 4.3% of 4,018 visits during the intervention period, yielding an average antimicrobial prescribing rate of 43 per 1,000 patient visits. Common indications for antimicrobial prescriptions included COPD exacerbation, community-acquired pneumonia, skin and soft-tissue infections, and herpes outbreaks.

Resident physicians were divided into low-intensity and high-intensity groups for the educational intervention based on their antimicrobial rate in the baseline period; 17 resident physicians were placed in the low-intensity group. All of these resident physicians (n = 17, 100%) received the low-intensity educational intervention. The high-intensity group included 20 resident physicians, of whom 12 (60%) received the entire high-intensity educational intervention; all received the low-intensity educational intervention.

**Fig. 1.**
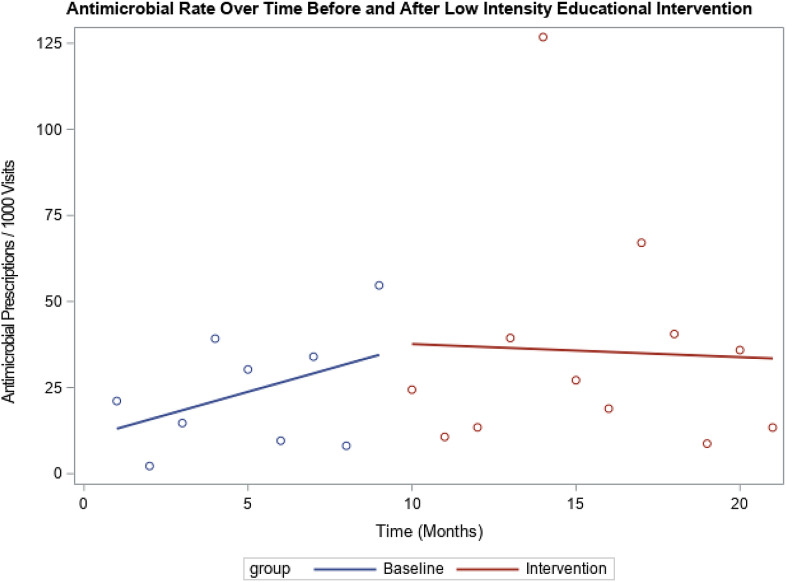
Antimicrobial rate over time before and after low intensity educational intervention. Comparison of trend in antimicrobial prescription rate among low-prescribing group before and after receiving low-intensity educational intervention. No statistically significant difference in the trends before and after intervention.

**Fig. 2.**
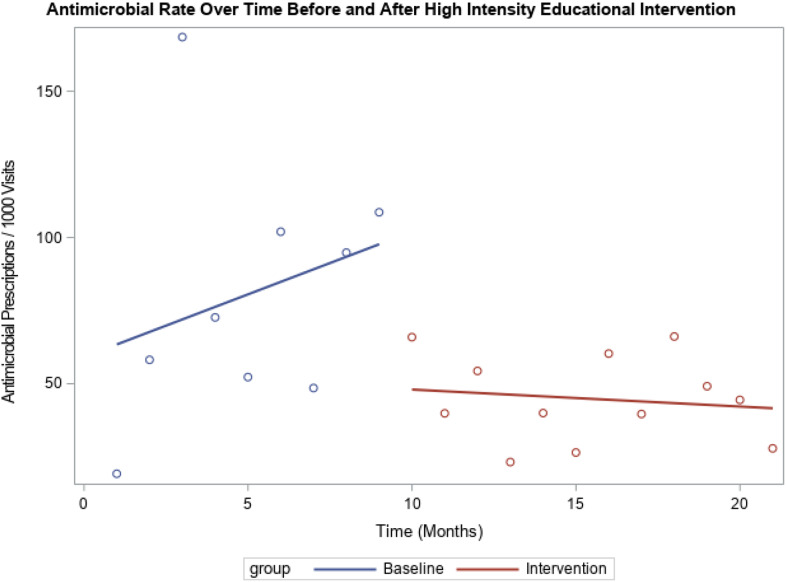
Antimicrobial rate over time before and after high intensity educational intervention. Comparison of trend in antimicrobial prescription rate among high-prescribing group before and after receiving high-intensity educational intervention. Statistically significant difference in the trends at baseline (increase by 3.46 prescriptions per 1,000 visits per month) and during the intervention (decreased by 2.04 prescriptions per 1,000 visits per month) (*P* = .05).

During the preintervention period (9 months), antimicrobial prescribing increased by 3.46 prescriptions per 1,000 visit per month for providers in the high-intensity educational intervention. During the intervention (12 months), there was a decrease of 2.04 prescriptions per 1,000 visits per month. The difference in prescribing trends between the preintervention versus the intervention period was statistically significant (*P* = .05), but the overall numbers of prescriptions were similar. For providers in the low-intensity education intervention, prescribing trends before the intervention versus during the intervention were not significantly different (2.07 vs 1.27; *P* = .62). The prescribing trends prior to the intervention were different between the high-intensity and low-intensity groups (*P* < .01). During the intervention, there was no statistically significant difference between the groups (*P* = .31). Results are shown in Figures 1 and 2, respectively.

## Discussion

A targeted educational intervention including in-person academic detailing was associated with a decrease in antimicrobial prescribing among resident physicians who prescribed more antimicrobials than their peers. Among resident physicians who prescribed fewer antibiotics at baseline, a lower-intensity educational intervention was not associated with a change in antimicrobial prescribing.

Academic detailing with resident physicians regarding outpatient antimicrobial prescribing is challenging. The busy schedules of resident physicians make it difficult to coordinate in-person discussions. Because of the low volume of acute infections seen in the continuity clinic, an exemplary or constructive case specific to the resident physician could not always be identified for discussion. Finally, there was a significant delay, often up to months, between the time that a prescription was given and feedback was discussed in the academic detailing session. Despite these challenges, our study shows that antimicrobial stewardship interventions can be successfully conducted with resident physicians.

This study had several limitations. It was small in size and was conducted at a single site. Without randomization and a control group, several additional variables may influence the results, including seasonality of respiratory viruses, differences in attending–resident physician relationships, improvements in resident-physician prescribing during training, and patient-level variables. Also, unique aspects of antimicrobial prescribing at the MVAHCS might affect generalization to other areas, including multiple sites of care for acute infections (ie, clinic, urgent care, and emergency department) and the use of a computer decision support system, which is a point-of-care reference for guideline-concordant prescribing of antimicrobials that is embedded in order entry. Likely in part due to these limitations, baseline prescribing of resident physicians at the MVAHCS is lower than that reported nationally among ambulatory visits (4.3% vs 12.6%).^1,9^ In addition, 8 of 20 resident physicians assigned to the high-intervention group were unable to be contacted for in-person academic detailing, largely due to scheduling and graduation. These limitations likely led to the underestimation of the difference in prescribing following the intervention. In contrast, resident physicians with high baseline prescribing could have undergone regression to the mean, which would overestimate the effect of the intervention.

Future work regarding application of antimicrobial stewardship to resident physicians is needed, including examination of whether prescribing is appropriate or guideline concordant. Future work should also assess whether these interventions have a durable effect over time. This study was limited to 1 year of follow-up, and the intervention was not sustained following the study period because key personnel were no longer available.
